# Public Mental Health Approaches for Building Resilience in Communities

**DOI:** 10.1192/j.eurpsy.2022.111

**Published:** 2022-09-01

**Authors:** C. Jung-Sievers, M. Coenen

**Affiliations:** Institute for Medical Information Processing, Biometry and Epidemiology, Pettenkofer School Of Public Health, Munich, Germany

**Keywords:** Public Mental Health, mental health promotion, prevention

## Abstract

Crises such as the corona pandemic, but also climate change associated events such as extreme weather events are major stressors for people on an individual, but also on a population level. Such crises often lead to highly burdened individuals with reduced quality of life, decreased well-being, mental health problems and an accumulation of psychiatric illnesses, especially in vulnerable population groups. These create a high demand and need for low intensive (psychosocial) support and primary and clinical care that can often no longer be adequately met by the existing infrastructure. However, good mental health and mental health care is a necessary prerequisite for people to lead fulfilling and productive lives and for communities and their settings (such as families, schools and workplaces, etc.) to function well. Therefore efficient supporting (public mental health) approaches are urgently needed. This presentation will introduce and discuss public mental health approaches and their effectiveness with a focus on mental health promotion and prevention. The implementation and dissemination of these approaches may help to further strengthen psychological resilience in communities to be better prepared for coping with acute crises and long-term stressors.

**Disclosure:**

No significant relationships.

